# Does economic policy uncertainty undermine stability of agricultural imports? Evidence from China

**DOI:** 10.1371/journal.pone.0265279

**Published:** 2022-03-15

**Authors:** Zhaohua Zhang, Roshini Brizmohun, Gang Li, Ping Wang

**Affiliations:** 1 College of Economics and Management, Shandong Agricultural University, Tai’an, China; 2 Faculty of Agriculture, University of Mauritius, Reduit, Mauritius; 3 School of Economics, Huazhong University of Science and Technology, Wuhan, China; 4 School of Business and Administration, Zhongnan University of Economics and Law, Wuhan, China; Universiti Malaysia Sabah, MALAYSIA

## Abstract

China is the world’s largest importer of agricultural products. Stability of agricultural imports directly affects domestic food availability, and hence influences national food security. This study is important to gauge effects of uncertainty resulting from global and domestic economic policy changes on the stability component of food security in China. Though many studies have explored the determinants and consequences of Chinese agricultural trade, research focusing on stability of agricultural imports is lacking. To fill the gap, this study calculates duration length and survival probability of China’s agri-food imports, and estimates effects of economic policy uncertainty (EPU) on the stability. Results show that trade duration of the agri-food imports is 12.07 months in China. However, 51.69% of disrupted trade relationships would resume after 2 months and 92.68% of temporarily interrupted trade relationships return to the market after 12 months. Empirical estimations show that global EPU has a larger impact on the stability of agricultural imports than Chinese EPU. Although Chinese EPU has heterogeneous effects on imports of different agri-food products in China, global EPU does not. Stabilized domestic food price and improved domestic agricultural productivity would improve stability of the imports significantly. The study concludes that China’s agricultural imports are less dynamic than previous studies claimed. However, EPU significantly erodes the trade stability. To offset negative effects of EPU on the stability, government should pay more attention on stabilizing domestic food price volatility and increasing food productivity, and therefore improve food security in China.

## 1. Introduction

In 2019, China imported USD 133.1 billion agricultural products, and became the largest importer of agricultural products in the world, surpassing the EU and the U.S. [1]. The increase in the agricultural and food imports over the last two decades is two-folds. Firstly, China has had to increase food imports to feed 1.44 billion people (18.46% of world’s population) with only limited cultivated land (10% of world’s cultivated land) [2]. Secondly, the growing disposable household income, rapidly urbanization, and changes of consumers’ preference have led to more demand for agri-food imports in China. The ratio of food imports over merchandise imports has fluctuated in China, rising from 4.6% in 2010 to 6.6% in 2019 [3] and this trend is projected to continue in the future [1, 2, 4]. Consequently, the nature of food security is changing as China’s agricultural imports overtake its agricultural exports.

Understanding the impact of global and domestic economic policy changes on agricultural and food trade will help policymakers take appropriate measures for a sustainable agricultural and food market and therefore ensure food security in China. Many studies have investigated the determinants and consequences of China’s large agricultural trading volume. However, the stability of agri-food imports, which may be viewed as a component of the FAO definition of food security, directly affects domestic food products availability and consumers’ food consumption, has not been sufficiently discussed. Huang et al. have argued that trade flow of agricultural imports depends heavily on government’s policy [4]. This paper therefore applies duration- and survival-techniques to investigate the important concept of stability of agricultural imports and explores to what extent economic policy uncertainty (EPU) affects agri-food import in China.

China’s agricultural imports grew at a much faster pace than exports over years. Statistics from International Trade Center (ITC) showed that the monthly agricultural exports of China increased from USD 2.33 billion in January 2005 to USD 6.06 billion in July 2020, while that of imports increased from USD 2.13 billion to 14.8 billion USD over the same period. The increasing trade volume implies that China plays a more important role in the world agricultural market, potentially influencing prices of the global market and stakeholders’ profit. Studies have validated the importance of agricultural imports to increase domestic agri-food availability and ensure food security [1, 5, 6]. However, a successful trade pattern or trade growth depends not only on exploring new markets and building new trade relationships, but also on the survival and growth of existing trade activities [7]. Previous studies were mainly focused on the impacts of agricultural trade on economic development in China [8, 9], but ignored the stability of agricultural imports. This study therefore aims to examine stability of China’s agri-food imports and quantify effects of EPU on the stability.

Trade duration measures the time period (i.e. years, months or days) for a trade activity that continuously exists [10, 11]. Generally, imported agri-food products supplement the domestic supply. Any failure or termination of agricultural trade activity reduces the trade duration and greatly affects the availability of agri-food products in domestic market. Trade duration study provides an effective tool to measure the trade pattern from a sustainability perspective—a longer trade duration indicates a more stable and sustainable trade relationship.

There are many determinants of trade duration, and among them market uncertainty is a newly discovered parameter that has received much attention recently. Market uncertainty arises from economic recession and/or government’s economic policies [12, 13]. During the COVID-19 pandemic, market uncertainty rose remarkably. Responding to the pandemic, most countries imposed strict city-, state- and nation-wide lockdown which dramatically interrupted international trade and global supply chain [14]. The aggregate international trade volume might have dropped as much as 32% in 2020 [15]. Under current situation of changing economic policies, it is of great practical significance to explore how trade duration and failure probability of trade activities would respond to the increased market uncertainty.

EPU index is extensively applied in existing studies to measure market uncertainty. Based on the frequency of news coverage on policies related to economic and/or market uncertainty, Baker et al. [12] developed a national EPU index for major economic entities, including an index for China (China_EPU). Based on the national EPU indices, Davis [16] constructed a monthly global EPU index (Global_EPU). The Global_EPU is a GDP weighted average of national EPU indices of 16 countries that accounts for two-thirds of global output. Recently, the ongoing trade conflicts between the U.S. and China coupled with the COVID-19 pandemic certainly increased both the magnitude and the volatility of the indices. The rapid increase in the indices is another motivation for this study.

In summary, China’s agricultural imports are growing at a fast pace and this trend is projected to continue in the future. It is assumed that the increasing EPU could undermine agricultural trade activities. This study first calculates the length of trade duration and intervals between trade spells for China’s agri-food imports, then it estimates the impacts of EPU on stability of the trade relationship. This is the first study that applies duration- and survival- techniques to examine trade patterns and its determinants of China’s agri-food imports. Based on the empirical results, this study provides policy suggestions to stabilize agri-food products’ market and improve food security in China.

This study makes contributions to three strands of literature. First, compared to previous study on trade dynamics, this study applies the latest monthly comprehensive dataset and unveils a novel picture of China’s agricultural imports. Second, it enriches studies on national food security from the perspective of food imports stability. Third, it extends research on EPU by quantifying its effects on stability of agri-food imports in China. The remaining study is arranged as follows: literature review on studies of trade patterns and economic influences of EPU are presented in section 2; the data description is in section 3; methodology and empirical estimation results are shown in section 4; the conclusions are summarized in section 5; and section 6 states the policy implications.

## 2. Literature review

Agricultural trade is considered extremely important as it is a fundamental element to alleviate poverty, improve food security, and also achieve the Sustainable Development Goals set by the United Nations [6, 17]. For example, Brooks and Matthews [18] presented in their research work that agricultural trade improved every dimension of food security, i.e. food availability, stability, utility, and accessibility, by extending and stabilizing domestic food supply, increasing household income, and improving food quality and nutrition knowledge. However, few study examines stability of China’s agricultural imports.

Classic trade theory and other empirical studies tend to explain why a particular trade activity exists or demonstrate the consequences of international trade [19–21]. However, these studies ignored stability or failure probability of a trade activity. In their pioneering work, Besedeš and Prusa [10] surprisingly discovered that an average trade duration of aggregate US imports only lasted for one to four years. More empirical investigations in other developed countries have been done. For example, Sabuhoro et al. [22] investigated export duration of Canadian products and found that the median survival time was only 20 months. Subsequently, following the same analysis technique, extensive research has been done to examine duration of merchandise or service trade in European countries. Nitsch [23] discovered that the mean trade duration for German aggregate imports was about three years. Similarly, for the EU, the mean trade duration for their aggregate imports from 140 non-EU exporters was also about three years [24]. Trade pattern has been more sustainable in Asian countries than other western developed ones. Obashi [25] reported that the trade duration was about 7.7 years for intra-Asian machinery trade. Brenton et al. [7] claimed that disruption to a trade activity was very perilous in low-income countries and suggested that learning-by-doing was an effective way to increase the chance of export.

Agri-food products have some unique characteristics, such as the importance to influence food and nutrition security, the role to determine livelihood of disadvantaged people, and the perishability and seasonality. Trade pattern for agri-food products, therefore, is believed to be different from other commercial products, and deserve a separate study other than the analysis of aggregate commodity. Peterson et al. [26] analyzed the trade relationship of fresh fruit and vegetables between the U.S. and exporters. They reported that 55% of the trade relationships lasted less than three years, and about one third of them survived only for one year. Focusing on dairy exports duration and survival probability in New Zealand, Luo and Bano [27] found that around 50% of trade relationships survived only for about 2 years. Some research has been conducted on trade duration of agri-food products in developing countries. Wang et al. [28] studied trade duration of ASEAN’s seafood exports, and concluded that the average trade duration was 4.4 years over the study period. While the mean duration of Zambia’s agricultural products was only 1.7 years between 1996 and 2019 [29]. Idris et al. [30] found that, from 2000 to 2017, the median export survival time of processed food in Malaysia was two years. While a large body of literature examined agricultural trade pattern for different countries, few of them investigated the trade dynamics of China’s agri-food products imports.

Besides calculation of trade duration length, existing studies also estimated effects of factors on failure probability of trade activities. A higher failure probability indicates a lower stability of trade relationship. The economic development (i.e., GDP per capita), agricultural employment and productivity, government subsidies and taxes, sanitary and phytosanitary policies, trade volume, and other gravity variables (i.e., distance, border continuity, language) have been confirmed to affect the likelihood of trade failure significantly [22, 28, 31, 32]. Government policy changes certainly would affect market behavior and trade flow [4]. The EPU, which measures market risks induced from economic policies, would clearly alarm sustainable economic and social development, including international trade activities [33, 34]. Empirical research on effects of EPU on trade failure probability extends studies on trade pattern and provides an effective tool to analyze sustainability and stability of international trade.

Effects of EPU have gained much more attention recently due to its sharp increase in both magnitude and volatility. Several studies have confirmed that EPU has negative effects on firms’ research and investment, employment, consumer behavior, stock market performance, crude oil price, exchange rate, global trade growth, etc. [35–42]. The on-going COVID-19 pandemic raises global economic uncertainty, leading to agricultural trade disruption which raises food prices and reduces food security globally [43, 44]. Abaidoo [45] concluded that EPU arising from the U.S. and the Chinese economies have significant constraining impact on international trade. As China is taking more market share of the global market, researchers and policy makers are curious about whether China’s influence on international market will grow as well. Tam [46] showed that EPU of both the U.S. and China significantly affected global trade flow, however, they affected global market in different ways. Specifically, the impacts of the U.S. were largely attributed to its indirect trade linkages with other economies, while China influences global trade flow through direct integration into the global value chain. Moreover, Zhang et al. [47] applied EPU of the U.S. and EPU of China separately to estimate their effects on international economy markets, including stock performance, credit, energy, and commodity market. They reported that, although China had a growing influence on international market, the U.S. had a dominant power in all markets. Handley and Limão [48] studied the effects of China as a WTO member on both China’s economic performance and other markets. They concluded that joining the WTO reduced market uncertainty for China dramatically, increased China’s exports to the U.S. but not to the other markets, and raised the U.S. consumers’ income by an equivalent of a 13-percentage-point permanent tariff decrease.

Importantly, some research has analyzed effects of EPU on agricultural trade. Xiao et al. [49] concluded that China’s EPU affected agricultural products’ markets differently. Specifically, China’s EPU has a greater influence on future prices of maize and soybean than that of wheat. Based on a Time-Varying Parameter Stochastic Volatility Vector Autoregression model, Li and Li [50] confirmed that China’s EPU has a significant negative impact on sustainability of its net grain imports. However, they failed to quantify the effects.

Although previous research studied patterns of agricultural trade and estimated effects of EPU on economic development from various perspectives, to the best of our knowledge, few studies examined the stability of China’s agri-food imports and quantified the effects of EPU on the stability. This study aims to fill this gap.

## 3. Data description

For this study, the authors applied Chinese monthly agricultural imports data from the ITC statistics. The dataset consists of 187 months (January 2005-July 2020), 218 exporters, and 23 agri-food products based on two-digit Harmonized System code (chapters 01–23). Following previous studies, this study defines exporter-product as a trade relationship [26, 27], i.e., one specific agricultural product is imported from one specific exporter. A trade spell is defined as the period of time which a trade relationship occurs without any interruptions [26]. The duration of trade spell (trade duration) is defined as the consecutive number of months with positive imports [11, 26]. A trade activity with a longer duration is considered more stable and sustainable.

**Fig 1:**
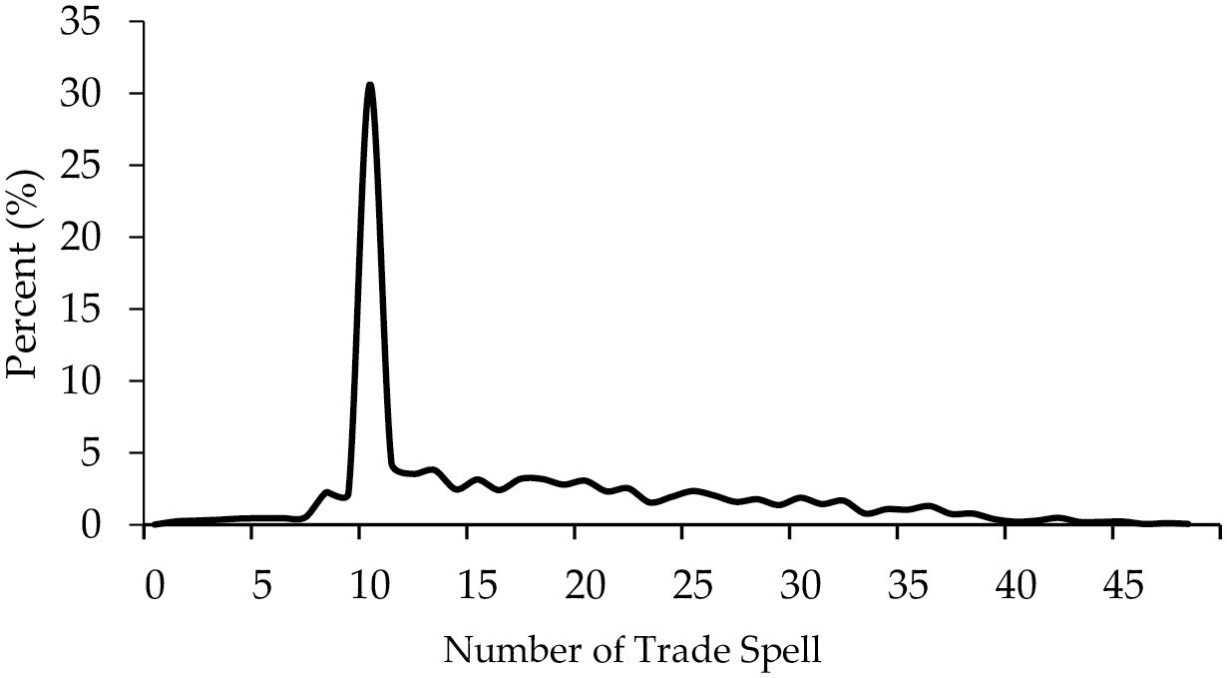
Distribution of trade spells. Data Source: Authors’ calculation.

The dataset has 2,547 trade relationships and 169,567 positive trade activities. The majority of trade relationships have more than one trade spell. This study considers multiple spells as an individual trade spell. Let us take China’s soybean imports in 2005 as an example. If the imports relationship was consecutively active from January 2005 to May 2005, but in June 2005 the trade activity stopped, and the trade activity resumed in July 2005 and continually lasted to December 2005, this study considers that there are two trade spells in 2005 with 5 months’ and 6 months’ trade duration, respectively. Fig 1 presents distribution of trade spells for this study. The number of trade spells per trade relationship varies remarkably during the research period. The number of trade spells has a mean of 17.22, and the median is 14. Only 383 (0.23%) trade activities have one trade spell, while 100 (0.06%) trade activities have 55 trade spells. And 30.62% of trade activities have 10 trade spells, the highest frequency of samples.

The average trade duration length of agri-food imports was 12.07 months in China (Table 3), similar to conclusions from previous studies [11, 26–29], indicating that the agricultural trade is highly dynamic. Table 1 presents distribution of the trade duration length. The median trade duration length was 15 months. And the longest trade duration of China’s agri-food imports was 19 months, accounting for only 0.65% of trade activities. The shortest trade duration was only one month, and they accounted for 8.94% of the trade activities. The majority of the trade activity (46.01%) lasted for 18 months over the study period, while no trade activities survived more than 19 months.

**Table 1:** Length of trade relationship (Source: Authors’ calculation).

Length (months)	Trade Activities	Frequency %
1	15,160	8.94
2	9,216	5.44
3	6,885	4.06
4	5,752	3.39
5	8,695	5.13
6	4,050	2.39
7	3,815	2.25
8	3,632	2.14
9	3,285	1.94
10	2,870	1.69
11	2,024	1.19
12	11,424	6.74
13	1,937	1.14
14	1,610	0.95
15	7,410	4.37
16	1,328	0.78
17	1,360	0.80
18	78,012	46.01
19	1,102	0.65
Total	169567	100

Besides length of trade duration, for the same trade relationship, this study also examines intervals between two trade spells to reflect trade dynamics. Shorter interval between trade spells is consider as a more stable and sustainable trade relationship. Table 2 presents the longevity before disrupted trade relationship resumes. The shortest interval is 2 months, while the longest interval is 179 months (about 15 years). And 51.69% of disrupted trade relationships resume after 2 months, while 92.68% of temporarily interrupted trade relationships are back to market after 12 months (one year) of disruption. Although Table 1 indicates that China’s agricultural imports are highly dynamic, Table 2 shows that most of China’s agricultural trade relationships resume after a short muted period, indicating the trade is relative stable.

**Table 2:** Intervals between two trade spells (Source: Authors’ calculation).

Interval (months)	Percent	Cumulating
2	51.69	51.69
3	16.79	68.49
4	7.66	76.15
5	4.68	80.83
6	3.38	84.22
7	2.37	86.59
8	1.9	88.49
9	1.5	89.99
10	1.07	91.06
11	1	92.07
12	0.79	92.86
…		
179	0.00	100

**Note**. The interval between two trade relationships is measured in month.

To quantify the effects of EPU on stability of China’s agricultural imports, this study applies the EPU index of China calculated by Davis et al. [13]. The EPU of China (China_EPU) reflects a scaled frequency count of articles about policy-related economic uncertainty in two mainland newspapers: Renmin Daily and Guangming Daily. The Remin Daily is the largest newspaper in China and Guangming Daily is a national daily newspaper targeting the higher educated groups. Davis [16] calculated a global EPU index to measure global market uncertainty (Global_EPU). These two indices reflect different sources of market uncertainty that alerts China’s agricultural imports. As China became the largest agricultural importer, it is important to examine whether China has an increasing market influence as well. This study applies the China_EPU and Global_EPU respectively, to distinguish and compare domestic and international influences. Table 3 presents statistic summary of the EPU indexes. Over given period, from January 2005 to July 2020, the mean of China_EPU is 153.31, while the mean of Global_EPU is 148.21. The China_EPU has always been higher than Global_EPU over the given period, and the maximum of China_EPU (649.07) is more than 50% larger than that of Global_EPU (429.46). Standard deviation of China_EPU is larger than that of Global_EPU as well, indicating that China_EPU has a higher volatility. Substantial volatility of EPU indices urge a comprehensive study of EPU’s influences on economic development, especially its effects on agricultural trade pattern of China.

**Table 3:** Statistics summary of determinants on agri-food imports.

Variable	Mean	Std. Dev.	Min	Max
Length	12.07	6.62	1	19
Spells	17.22	9.03	0	55
Intervals	4.36	10.22	1	178
China_EPU	153.31	108.18	23.72	649.07
Global_EPU	148.21	70.66	48.9	429.6
L_tradevalue	12.49	2.81	6.91	22.31
China_CPI	116.56	24.85	68.7	169.05
Foodcapita	94.92	7.14	80.85	102.81
China_urbanization	52.46	5.45	42.52	60.31
L_distance	8.76	0.72	6.7	9.87
Contiguity	0.1	0.3	0	1
L_GDP_o	19.64	1.89	10.55	223.79
L_GDP_d	22.67	0.61	21.54	23.39
WTO_exporter	0.93	0.25	0	1
RTA_type	1.13	0.47	0	2
RTA_coverage	0.42	0.79	0	2

Besides the EPU indices, Table 3 presents other determinants of agri-food product imports in China. Larger trade volume indicates a bigger market and larger initial investment. Any termination of trade relationship with a larger trade volume would cause more loss. The variable L_tradevalue is logarithm of bilateral trade value. The average L_tradevalue is 12.49 over the study period. Other demand and supply shifters of agri-food products also influence trade pattern of agricultural imports.

Domestic food prices influence food demand, which might affect trade flow of agri-food imports. Higher domestic food price will reduce demand of agri-food products, especially the non-necessities, which would increase the chance of agri-food imports failure. This study uses Consumer Prices Index (CPI) to measure domestic market price changes of food products. The variable China_CPI is monthly CPI of food (including food demand of restaurants) in China. Based on data released by Federal Reserve Economic Data of the U.S. and National Bureau of Statistics of China, the average value of China_CPI is 116.56 over the study period. China_CPI has a wide variation, and the largest value of CPI is more than two times of the smallest value.

Urbanization ratio (China_urbanization) measures proportion of population who live in urban area in China. Compared to rural residents, urban residents have higher living standard and greater purchasing power for high quality food products. Therefore, higher level of urbanization ratio indicates higher demand for high-quality imported food products, which might stabilize agri-food imports. Domestic food productivity affects agri-food imports by changing domestic agri-food products supply. With a higher productivity, more products would be provided with the same level of inputs, which would decrease the need for imports. Change in domestic food productivity is captured by Foodcapita which is an index of gross per capita food production. Mean of Foodcapita is 94.92, and its maximum value is 102.81.

Other explanatory variables are motivated by classic gravity model analysis. All gravity variables are collected from the database of Centre d’Etudes Prospectives et d’Informations Internationales (CEPII). L_distance is a logarithm of the distance between China and its exporter, which measures the most populous city in each country in kilo metre. Variable Contiguity is a dummy variable that indicates whether an exporter shares border with China. Higher Gross Domestic Product (GDP) means a bigger market and a higher purchasing power, and therefore, more demand for agri-food products imports. This study applies logarithm of GDP, L_GDP_o and L_GDP_d, to represent economic development of exporter and China, respectively.

The WTO sets rules for the members, for example market transparency requirement and the most-favoured-nation treatment. This study assumes that having a trade relationship with a WTO member (WTO_exporter) would decrease the probability of a trade failure, hence, increase trade stability. Furthermore, trade negotiations and agreements help to regulate trade relationships and stabilize trade market. Different trade agreement types (TRA_type) or coverage (TRA_coverage) might have different impacts on the stability.

## 4. Methodology and empirical estimations

Survival analysis techniques are extensively applied in examining stability of international trade [10, 22, 23, 26–28]. The survival analysis consists of parametric (Cox proportional hazard models) and non-parametric (Kaplan-Meier) estimators. Although many studies have proved the robustness of Kaplan-Meier (KM) estimator to censored data, this is not the case for continuous Cox proportional hazard models. Hess and Persson [24] questioned the efficiency of the Cox proportional hazard model while applied to a large trade dataset. International trade data do not always hold the assumption of proportional hazard assumption that the Cox model requires [28], and the Cox model fails to address tied trade spells and heterogeneity issues. Following recent practice, this study applies KM estimator and a discrete-time model to calculate trade duration length and estimate impacts of influencing factors on stability of agricultural imports in China.

### 4.1. Model description

The KM estimator calculates the number of observations that survives over the total number of observations that are at risk in the given period *t* [10], a higher survival rate (*S*_*it*_) indicates a higher stability of the imports:

$$S_{it}=\prod_{t_{i}\leq t}\frac{n_{i}-f_{i}}{n_{i}}$$ (1)

Though the KM estimator is effective to make pair-wise comparisons, it could not investigate and quantify impacts of influencing factors on the stability. A discrete survival model is applied to quantify the impacts. Let *T*_*i*_ be a non-negative random variable that measures the survival time of the *i*^*th*^ trade spell. The discrete time intervals can be shown as [*t*_1_, *t*_2_, …, *t*_*max*_]. Besedeš and Prusa [10] define the survival function for a trade spell at a random variable *T*_*i*_ is shown in the following equation:

$$S_{it}=P(T_{i}>t_{i})=\sum_{t_{j}>t}p(t_{j})$$ (2)

The probability that *i*^th^ trade spell ends in *t*^th^ time interval is called hazard probability. It is conditional on the relationship surviving up to the beginning of that time interval, and on other determinants. Eq 3 shows the hazard probability defined by Hess and Persson [51]:

$$h_{it}=P(T_{j}<t_{t+1}|T_{j}>t_{t},\;X_{it})$$ (3)

where 0 ≤h_it≤1, and ***X***_*it*_ is a vector of covariates that impacts hazard probability. Assume *stop*_*it*_ is a binary variable to indicate whether a trade spell fails at time *t*, this study will apply a probit model to examine the effects of covariates on failure probability of the imports (Eq 3), a higher failure probability indicates a lower stability of trade activity.


$$probit({stop}_{it})=f(X_{it})=f(EPU,\;trade\;value,\;demand,\;gravity)$$ (4)

Covariates include EPU indices (China_EPU and Global_EPU), initial trade value of the trade spell (L_tradevalue), a vector of demand shifters and a vector of gravity variables. The demand shifters vector includes domestic food price volatility (China_CPI), urbanization level of China (China_urbanization), and domestic food productivity per capita index (Foodcapita). Gravity variables vector includes distance between exporters and China (L_distance), contiguity between China and exporter (Contiguity), gross domestic products of both exporters and China (L_GDP_o and L_GDP_d), WTO membership of exporters (WTO_exporter), and bilateral regional trade agreement type (RTA_type) and its coverage (RTA_coverage).

### 4.2. Kaplan-Meier estimator

**Fig 2:**
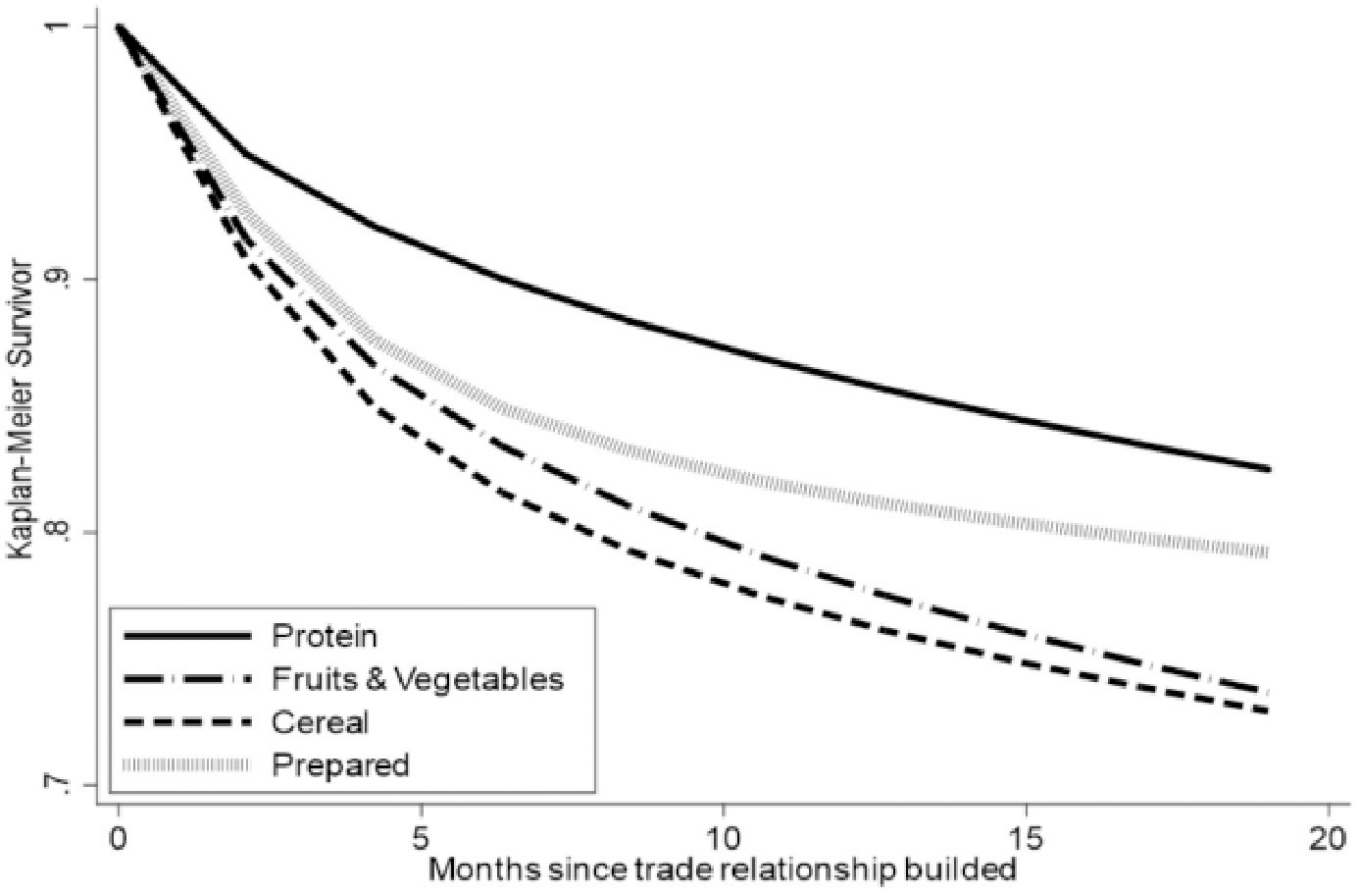
Kaplan-Meier estimator for agri-food products.

The KM estimators for different categories of agri-food products are presented in Fig 2. For all agri-food products, the survival rate of trade activities drops dramatically after the first month, and it decreases at a slower pace in the consecutive months with no trade activity surviving more than 19 months. Among all four categories of agri-food products, products that provide major protein source has the most stable relationship with the highest survival rate, while cereals are most dynamic with the lowest survival rate. The survival rate difference between imported fruits and vegetables and imported cereals is smaller than that between other agri-food groups.

Due to limited data availability, this study covers China’s agricultural imports activities between January 2005 and July 2020. If a trade spell is active in January 2005, it is unknown whether the trade spell started before the starting period (January 2005). Similarly, whether the active trade spell in July 2020 would continue to exist in the following month (August 2020) is not known. Therefore, left censored issue occurs when trade spells started before January 2005, and right censored issue happens as trade spells continue past July 2020. Compared to annual data, monthly data have less censored observations. For example, there are 20% censored observations in Besedeš and Prusa [10] and about 40% censored observations in Peterson et al. [26], while this study only has 0.4% left censored observations and 0.67% right censored observations. Hess and Persson [24] claimed that the right censored observations did not affect estimation efficiency while left censored observations might cause estimation bias, and therefore suggested that all left censored observation should be dropped. Following previous practice [24, 28], this study drops all left censored observations. To examine how left censored observations affect KM estimator, results of both left censored dropped sub-sample and whole sample are presented in Table 4.

**Table 4:** Kaplan-Meier estimators of China’s agricultural imports.

		Trade Duration (months)	Estimated KM Survival Rate				
			1^st^	6^th^	12^th^	18^th^	19^th^
**Whole Sample**		12.07	0.91	0.85	0.83	0.78	0.74
**Left Censored Dropped Sample**		12.12	0.91	0.85	0.83	0.78	0.74
**Category**	*Protein*	13.46	0.94	0.90	0.88	0.82	--
	*Fruits & Vegetable*	10.66	0.91	0.83	0.80	0.74	--
	*Cereal*	10.89	0.90	0.81	0.79	0.74	--
	*Prepared*	12.58	0.91	0.86	0.84	0.79	0.75
**WTO**	=1	12.38	0.91	0.85	0.83	0.78	0.74
	=0	8.24	0.81	0.70	0.67	0.62	0.59

**Note**.—means do not apply.

Although the average value of trade duration length is slightly higher when the left censored samples are dropped, survival rates of both whole sample and sub-sample at a specific time do not show any difference (Table 4). This study applies whole sample to study heterogeneity of trade duration among different agri-food products. The protein sources products have the longest trade duration (13.46 months), followed by prepared products (12.58 months), cereals (10.89 months), and fruit and vegetable (10.66 months). Though protein products have the highest survival rate at any given specific time, only the prepared products have active trade activity in the 19^th^ month.

Researchers and policy makers consistently agree that the WTO has made a great contribution to eliminate trade barriers. The WTO regulations are assumed to affect trade duration and survival rate of China’s agricultural imports as well. Table 4 presents China’s agricultural imports’ trade duration and survival probability at a specific time considering whether the exporter is a WTO member. Bilateral trade relationships with WTO members clearly increase trade duration length. The trade duration is 12.38 months if the exporter is a member of WTO. On the other hand, with a non-WTO member the trade duration is only 8.24 months. Building a trade relationship with a member of WTO increases trade duration length by at least 50%.

### 4.3 Estimation results of the discrete model

Coefficients estimation of Eq 4 and the corresponding marginal effects of explanatory variables are presented in Table 5. This study applied two models by including either China_EPU or Global_EPU as a main determinant of the stability of China’s agri-food products imports. Both domestic economic policy uncertainty (China_EPU) and international economic policy uncertainty (Global_EPU) have a significantly positive effect on the stability. However, the marginal effect of the Global_EPU is larger than that of China_EPU. Another one score higher of Global_EPU index would increase the failure probability by 0.03%, while one score increase of China_EPU would raise it by 0.01%. It indicates that, compared to market influence induced from international EPU, China’s influences on its agricultural imports are smaller.

**Table 5:** Empirical results of discrete survival model (Eq 4).

	Coefficient	M.E^1^	Coefficient	M.E^1^
China_EPU	0.0003**	0.0001**	--	--
Global_EPU	--	--	0.002***	0.0003***
L_tradevalue	-0.01***	-0.002***	-0.01***	-0.002***
Length	-0.125***	-0.025***	-0.125***	-0.025***
China_CPI	-0.0001	-0.00001	-0.003*	-0.001**
Foodcapita	0.031***	0.006***	0.039***	0.008***
Urbanization	-0.038***	-0.008***	-0.046***	-0.009***
L_distance	-0.011	-0.002	-0.011	-0.002
Contiguity	-0.091***	-0.018***	-0.091***	-0.018***
L_gdp_o	0.0004*	0.0001*	0.0004*	0.0001*
L_gdp_d	-0.005***	-0.001***	-0.005***	-0.001***
WTO_o	-0.026	-0.005	-0.026	-0.005
RTA_type	-0.03**	-0.006**	-0.03**	-0.006**
TRA_coverage	0.029***	0.006***	0.029***	0.006***
Intercept	-.227		-.465**	

Note.

^1^ M.E indicates marginal effect and—means do not apply.

*** is 99% significant.

** is 95% significant.

* is 90% significant.

Interrupted trade relationship that has a larger trade volume results in larger trade costs to traders. For example, companies involved in a large trade volume will lose their huge initial investment if they leave the market, and therefore trade partners tend to stabilize and extend trade relationship to minimize the costs. Estimation results show that trade value (L_tradevalue) has a significant negative effect on the failure probability. In another word, a high initial trade value decreases the risk that an imported agricultural relationship fails in China. Both models show that one percent increase of bilateral trade value decreases the failure probability by 0.2%. Length of a trade spell also significantly decreases the failure probability. Another month increase in trade spell length decreases the failure probability by 2.5% for both models.

Domestic food CPI index (China_CPI) has a significantly negative effect on the failure probability when the Global_EPU is applied. However, this effect is insignificant when Global_EPU is replaced by China_EPU. The last column of Table 4 shows that one score increase in China_CPI increases the probability of trade failure by 0.1%. Domestic food productivity (Foodcapita) has a positive effect on the failure probability of agricultural imports. The marginal effect of Foodcapita is larger for the Global_EPU model than for the model of China_EPU. One score higher of Foodcapita would increase the failure probability by 0.6% and 0.8% from the China_EPU and Global_EPU model, respectively. Urbanization level shows a negative effect on the probability of trade failure. Higher urbanization level means more food demand and higher living standards. One percent increase in urbanization rate would reduce the failure probability by 0.8% when China_EPU is applied, while that is 0.9% when the Global_EPU is applied.

Other gravity variables also affect the failure probability. Distance (L_distance) between exporters and China did not show significant effects for both models, while the variable Contiguity shows a significant negative effect. If the exporter shares boarder with China, the chance of trade failure decreases by 1.8%. Economic development also affects failure probability of bilateral trade relationship and China’s GDP has a larger impact than exporter’s GDP. One percent increase in China’s GDP would decrease the failure probability by 0.1%, compared to that of 0.01% decrease induced from exporter’s GDP. China’s rapid economic growth would increase living standards and require more high quality food, such as imported meat, dairy, or seafood products.

Bilateral trade agreements promote survival probability of bilateral trade significantly. They eliminate trade barriers, such as tariffs and imports quotas. As 93% of the exporters are WTO members, small variation of the variable possibly explains why the WTO membership does not show significant impact on the failure probability. The type of regional trade agreements (RTA_type) affects China’s agricultural imports activity significantly. Compared to Partial Scope Agreement (PSA), Free Trade Agreement or Economic Integration Agreement (FTA/EIA) has a larger impact. Commodity coverage of RTA (RTA_coverage) also affects the failure probability. The RTA which only covers commercial goods reduces the probability of trade relationship stop significantly as opposed to the RTA that covers both goods and services. Exporters that have RTA with China would reduce probability of trade interruption by 0.6%.

### 4.4 Heterogeneous effects of EPU among agri-food products

Besides quantifying effects of EPU and other influential factors on the stability of aggregate agri-food imports, another question that rises is whether EPU has heterogeneous effects on different agri-food products. This study divides whole sample into four sub-samples based on product characteristics, namely, protein, prepared, fruit and vegetable, and cereal products. For the sub-samples, this study repeats model estimation (Eq 4) and tests whether the differences among coefficients are significant. Table 6 only presents coefficients of China_EPU and Global_EPU for different agri-food products, the complete regression results are available in appendix (Table A.1 to Table A.4 in S1 Appendix).

**Table 6:** Effects of economic policy uncertainty on different agri-food products.

	Whole	Protein	Prepared	Fruit & Vegetable	Cereal
**China_EPU**	0.0002**	0.0005***	0.0007***	0.0004*	0.0009***
**F-test**	Chi2(4) = 15.11, Prob>chi2 = 0.005				
**t-test**		Chi2(1) = 3.72, Prob>chi2 = 0.05			
			Chi2(1) = 10.14 Prob>chi2 = 0.002		
				Chi2(1) = 0.99, Prob>chi2 = 0.32	
					Chi2(1) = 3.78, Prob>chi2 = 0.05
**Global_EPU**	0.001***	0.002***	0.002***	0.002***	0.001*
**F-test**	Chi2(4) = 6.03, Prob>chi2 = 0.2				
**t-test**		Chi2(1) = 3.9, Prob>chi2 = 0.05			
			Chi2(1) = 0.99, Prob>chi2 = 0.32		
				Chi2(1) = 0.91, Prob>chi2 = 0.34	
					Chi2(1) = 0.27, Prob>chi2 = 0.61

Note:

*** is 99% significant

** is 95% significant

* is 90% significant

Table 6 shows that both China_EPU and Global_EPU have a significant effect on all agri-food products. For all effects, significant differences among magnitudes indicate heterogeneous impacts of EPU on products. First, this study applies F-test to investigates whether there are magnitude differences of EPU effects among all groups. Second, it applies t-test to examine whether there is difference of EPU effects between whole sample and other sub-sample groups, for example the difference between whole-sample and sub-sample of protein products. With respect to effects of China_EPU, the F-test shows that there is significant difference among all groups, implying China_EPU has heterogeneous effects on different agri-food products. The t-test shows that, compared to the aggregate agri-food, China_EPU has a significant different effect on all sub-sample groups except fruit and vegetable products. However, for the Global_EPU, the F-test does not show significant results, implying that Global_EPU does not have heterogeneous effects on agri-food products.

## 5. Conclusions

This study applies duration- and survival-analysis technique to examine trade pattern of agri-food imports in China. On average, trade spell lasts for 12.07 months. Considering trade duration length alone, agricultural imports are highly dynamic which is consistent to previous finding [26, 27, 29]. However, previous studies did not take intervals between trade spells into consideration. In our sample, 30.62% of trade relationship has ten trade spells over the study period, while trade relationship has about 17.22 trade spells on average. Intervals between trade spells are very short. 51.69% of failed trade relationship would resume after two months, 84.22% of stopped trade relationship revives within six months (half a year), and 92.68% of temporarily interrupted trade relationships are back to market after 12 months (one year). Combining trade duration length and short intervals between two trade spells, this study concludes that agri-food imports are relative stable in China. The other conclusions are shown as followings:

1. Both Chinese EPU and global EPU decreases stability of the existing agricultural trade relationship significantly, while the global EPU index has a larger impact on Chinese agri-food imports than China’s EPU.
2. Effects of EPU indices on the stability vary among agri-food products. Protein-sourced food has a different trade pattern from others with a longer survival rate. The China_EPU has heterogeneity impacts on different agri-food products, while effects of Global_EPU do not have significant differences among all groups.
3. Domestic food price volatility has a significant impact on the stability for the model of Global_EPU, but the effect is not significant for model of China_EPU. The domestic food price volatility can offset negative effect of Global_EPU on stability of agri-food imports in China.
4. Kaplan-Meier estimators show that WTO membership extends trade duration length. And binary empirical estimation results confirm that regional trade agreements significantly increase the stability of agricultural imports in China, and therefore, extend trade duration length correspondingly.

## 6. Policy implications

Based on the conclusions, the study comes up with several policy implications to help China fight for food security. The study showed that global EPU index has a larger impact on Chinese agri-food imports than China’s EPU, indicating that China does not have enough market power and therefore cannot influence agricultural trade market. China’s agri-food imports can be easily affected by economic uncertainty induced from international market. With increasing dependency on international market for agri-food products supply, lack of market power might erode China’s food security and sustainable development in future. China government should pay more attention on mitigating agri-food price shocks from international market.

The China_EPU has heterogeneity impacts on different agri-food products, while effects of Global_EPU do not have significant differences among all groups. The results suggest that, to stabilize agri-food imports, China government could launch product-targeted policy. Effects of both China_EPU and Global_EPU on protein sourced products are significantly different from the whole sample. Kaplan-Meier indicates protein-sourced food has a different trade pattern from others with a longer survival rate. As consumers’ preference changes and dietary habit shifts from traditional staples and vegetables to high protein products, the importance of protein products for food security increases in China. Considering the production restrictions of high protein products, especially animal sources protein, government needs to pay more attention on stabilizing protein products imports.

Domestic food price volatility has a significant impact on the stability for the model of Global_EPU, but the effect is not significant for model of China_EPU. The domestic food price volatility can offset negative effect of Global_EPU on stability of agri-food imports in China. Government could offset uncertainty effects from international market through actively stabilizing domestic food price volatility. Domestic food productivity has a positive effect on the failure probability. Hence, if China improves its agricultural productivity, the likelihood of the imports stops would increase. Although economic uncertainty induced from international market could not be controlled, efforts to diminish negative effects from international market by increasing domestic productivity could be adopted, such as increase investment in agricultural related research, reduce small farmers’ access barriers to bank and other financial institutions.

To ensure international trade move smoothly and predictably, the WTO set a series of rules for bilateral trade. Kaplan-Meier estimators show that WTO membership extends trade duration length. And binary empirical estimation results confirm that regional trade agreements significantly increase the stability of agricultural imports in China, and therefore, extend trade duration length correspondingly. Furthermore, trade agreement that focuses on goods only has a larger impact on the stability. Importing agri-food products from a WTO member, especially those having a bilateral trade agreement with China, will help to stabilize agri-food imports and improve food security in China.

## Supporting information

S1 Appendix (DOCX)

## References

[1] United States Department of Agriculture. China: Evolving Demand in the World’s Largest Agricultural Import Market [Internet]. 2020. https://www.fas.usda.gov/sites/default/files/2020-09/china-iatr-2020-final.pdf

[2] Sheng Y, Song L. Agricultural production and food consumption in China: A long-term projection. China Economic Review. 2019;53:15–29.

[3] World Bank. China—Food imports as a share of merchandise imports [Internet]. 2020. https://knoema.com/atlas/China/Food-imports

[4] Huang J, Wei WEI, Qi CUI, Wei XIE. The prospects for China’s food security and imports: Will China starve the world via imports? Journal of integrative agriculture. 2017;16(12):2933–44.

[5] Diaz-Bonilla E. Agricultural trade and food security: some thoughts about a continuous debate. Strengthening the Global Trade System. 2013;39.

[6] Berg A, Krueger A. Trade, growth and poverty. Macroeconomic Policies and Poverty Reduction. 2002;70.

[7] Brenton P, Saborowski C, Von Uexkull E. What explains the low survival rate of developing country export flows? The World Bank Economic Review. 2010;24(3):474–99.

[8] Huang J, Yang J, Zhigang XU, Rozelle S, Ninghui LI. Agricultural trade liberalization and poverty in China. China Economic Review. 2007;18(3):244–65.

[9] Qiang W, Liu A, Cheng S, Kastner T, Xie G. Agricultural trade and virtual land use: the case of China’s crop trade. Land Use Policy. 2013;33:141–50.

[10] Besedeš T, Prusa TJ. Product differentiation and duration of US import trade. Journal of international Economics. 2006;70(2):339–58.

[11] Zhang D, Tveterås R. A fish out of water? Survival of seafood products from developing countries in the EU market. Marine Policy. 2019;103:50–8.

[12] Baker SR, Bloom N, Davis SJ. Measuring economic policy uncertainty. The quarterly journal of economics. 2016;131(4):1593–636.

[13] Davis SJ, Liu D, Sheng XS. Economic policy uncertainty in China since 1949: The view from mainland newspapers. Work Pap. 2019;1–35.

[14] Altig D, Baker S, Barrero J, Bloom N, Bunn P, Chen S, et al. Economic uncertainty in the wake of the COVID-19 pandemic. VoxEU org. 2020;24. 10.1016/j.jpubeco.2020.104274 PMC7480328 32921841

[15] World Trade Organization. Trade set to plunge as COVID-19 pandemic upends global economy. World Trade Organization Geneva; 2020.

[16] Davis SJ. An index of global economic policy uncertainty. National Bureau of Economic Research; 2016.

[17] Kanter DR, Musumba M, Wood SL, Palm C, Antle J, Balvanera P, et al. Evaluating agricultural trade-offs in the age of sustainable development. Agricultural Systems. 2018;163:73–88.

[18] Brooks J, Matthews A. Trade dimensions of food security. 2015;

[19] Grant JH, Lambert DM. Do regional trade agreements increase members’ agricultural trade? American journal of agricultural economics. 2008;90(3):765–82.

[20] Maertens M, Swinnen J. Agricultural trade and development: a value chain perspective. WTO Staff Working Paper; 2015.

[21] Grossman GM, Helpman E. Comparative advantage and long-run growth. National Bureau of Economic Research Cambridge, Mass., USA; 1989.

[22] Bosco Sabuhoro J, Larue B, Gervais Y. Factors determining the success or failure of Canadian establishments on foreign markets: A survival analysis approach. The International Trade Journal. 2006;20(1):33–73.

[23] Nitsch V. Die another day: Duration in German import trade. Review of World Economics. 2009;145(1):133–54.

[24] Hess W, Persson M. Exploring the duration of EU imports. Review of World Economics. 2011;147(4):665–92.

[25] Obashi A. Stability of production networks in East Asia: Duration and survival of trade. Japan and the World Economy. 2010;22(1):21–30.

[26] Peterson EB, Grant JH, Rudi-Polloshka J. Survival of the Fittest: Export Duration and Failure into United States Fresh Fruit and Vegetable Markets. American Journal of Agricultural Economics. 2018;100(1):23–45.

[27] Luo Y, Bano S. Modelling New Zealand dairy products: Evidence on export survival and duration. Australian Journal of Agricultural and Resource Economics. 2020;64(3):605–31.

[28] Wang P, Tran N, Wilson NLW, Chan CY, Dao D. An Analysis of Seafood Trade Duration: The Case of ASEAN. Marine Resource Economics. 2019;34(1):59–76.

[29] Phiri J, Malec K, Majune SK, Appiah-Kubi SNK, Gebeltová Z, Kotásková SK, et al. Durability of Zambia’s Agricultural Exports. Agriculture. 2021;11(1):73.

[30] Idris A, Ismail NW, Sidique SFA, Kaliappan SR. The Export Survival of Malaysia’s Processed Food. International Journal of Economics & Management. 2020;14(2).

[31] Bojnec Š, Fertő I. The duration of global agri-food export competitiveness. British Food Journal. 2017.

[32] Béné C, Fanzo J, Prager SD, Achicanoy HA, Mapes BR, Alvarez Toro P, et al. Global drivers of food system (un) sustainability: A multi-country correlation analysis. PloS one. 2020;15(4):e0231071. doi: 10.1371/journal.pone.0231071 32243471 PMC7122815

[33] Brogaard J, Detzel A. The asset-pricing implications of government economic policy uncertainty. Management Science. 2015;61(1):3–18.

[34] Kang W, Lee K, Ratti RA. Economic policy uncertainty and firm-level investment. Journal of Macroeconomics. 2014;39:42–53.

[35] Bloom N. Fluctuations in uncertainty. Journal of Economic Perspectives. 2014;28(2):153–76.

[36] Aloui R, Gupta R, Miller SM. Uncertainty and crude oil returns. Energy Economics. 2016;55:92–100.

[37] Schaal E. Uncertainty and unemployment. Econometrica. 2017;85(6):1675–721.

[38] Chen J, Jiang F, Tong G. Economic policy uncertainty in China and stock market expected returns. Accounting & Finance. 2017;57(5):1265–86.

[39] Chen L, Du Z, Hu Z. Impact of economic policy uncertainty on exchange rate volatility of China. Finance Research Letters. 2020;32:101266.

[40] Constantinescu C, Mattoo A, Ruta M. Policy uncertainty, trade and global value chains: some facts, many questions. Review of Industrial Organization. 2020;57(2):285–308.

[41] Caldara D, Iacoviello M, Molligo P, Prestipino A, Raffo A. The economic effects of trade policy uncertainty. Journal of Monetary Economics. 2020;109:38–59.

[42] Kirchner S. State of confusion: Economic policy uncertainty and international trade and investment. Australian Economic Review. 2019;52(2):178–99.

[43] Barichello R. The COVID-19 pandemic: Anticipating its effects on Canada’s agricultural trade. Canadian Journal of Agricultural Economics/Revue canadienne d’agroeconomie. 2020;68(2):219–24.

[44] Baldwin R, Freeman R. Trade conflict in the age of Covid-19. VoxEU org. 2021.

[45] Abaidoo R. Policy uncertainty and dynamics of international trade. Journal of Financial Economic Policy. 2019.

[46] Tam PS. Global trade flows and economic policy uncertainty. Applied Economics. 2018;50(34–35):3718–34.

[47] Zhang D, Lei L, Ji Q, Kutan AM. Economic policy uncertainty in the US and China and their impact on the global markets. Economic Modelling. 2019;79:47–56.

[48] Handley K, Limao N. Policy uncertainty, trade, and welfare: Theory and evidence for China and the United States. American Economic Review. 2017;107(9):2731–83.

[49] Xiao X, Tian Q, Hou S, Li C. Economic policy uncertainty and grain futures price volatility: evidence from China. China Agricultural Economic Review. 2019.

[50] Li Y, Li J. How Does China’s Economic Policy Uncertainty Affect the Sustainability of Its Net Grain Imports? Sustainability. 2021;13(12):6899.

[51] Hess W, Persson M. The duration of trade revisited. Empirical Economics. 2012;43(3):1083–107.

