# Supplementary material for: Does economic policy uncertainty undermine stability of agricultural imports? Evidence from China
Source: PLoS One. 2022 Mar 15;17(3):e0265279. doi: 10.1371/journal.pone.0265279 (PMC8923469; doi:10.1371/journal.pone.0265279)
Supplement: S1 Appendix — (DOCX) [file pone.0265279.s001.docx]

**Appendix A. Estimation results of equation 4 for sub-samples**

**Table A. 1 Estimation results of protein products**

|  |  | **Coefficients** | **M.E^1^** |  | **Coefficients** | **M.E^1^** |
| --- | --- | --- | --- | --- | --- | --- |
| China_EPU |  | 0.0003 | 0.0001 |  | -- | -- |
| Global_EPU |  | -- | -- |  | 0.002*** | 0.0003*** |
| L_tradevalue |  | 0.008 | 0.001 |  | 0.007 | 0.001 |
| Length |  | -0.126*** | -0.023*** |  | -0.126*** | -0.023*** |
| China_CPI |  | -0.002 | -0.0004 |  | -0.005* | -0.001* |
| Foodcapita |  | 0.051*** | 0.009*** |  | 0.058*** | 0.011*** |
| Urbanization |  | -0.055*** | -0.01*** |  | -0.062*** | -0.011*** |
| L_distance |  | 0.016 | 0.003 |  | 0.016 | 0.003 |
| Contiguity |  | -0.046 | -0.008 |  | -0.045 | -0.008 |
| L_gdp_o |  | -0.007 | -0.001 |  | -0.008 | -0.001 |
| L_gdp_d |  | -0.035* | -0.006* |  | -0.036* | -0.007* |
| WTO_o |  | 0.044 | 0.008 |  | 0.043 | 0.008 |
| RTA_type |  | -0.044 | -0.008 |  | -0.043 | -0.008 |
| TRA_coverage |  | 0.037 | 0.007 |  | 0.037 | 0.007 |
| Intercept |  | -0.52 |  |  | -0.708 |  |

**Note**. ^1^ M.E indicates marginal effect and -- means do not apply.

*** is 99% significant; ** is 95% significant; * is 90% significant

**Table A. 2** Estimation results of prepared products

|  |  | **Coefficients** | **M.E^1^** |  | **Coefficients** | **M.E^1^** |
| --- | --- | --- | --- | --- | --- | --- |
| China_EPU |  | 0.0004** | 0.0001** |  | -- | -- |
| Global_EPU |  | -- | -- |  | 0.001*** | 0.0002*** |
| L_tradevalue |  | 0.02*** | 0.004*** |  | 0.019*** | 0.004*** |
| Length |  | -0.13*** | -0.024*** |  | -0.13*** | -0.024*** |
| China_CPI |  | 0.001 | 0.0002 |  | -0.001 | -0.0003 |
| Foodcapita |  | 0.044*** | 0.008*** |  | 0.046*** | 0.009*** |
| Urbanization |  | -0.066*** | -0.012*** |  | -0.061*** | -0.011*** |
| L_distance |  | -0.048** | -0.009** |  | -0.048** | -0.009** |
| Contiguity |  | -0.039 | -0.007 |  | -0.039 | -0.007 |
| L_gdp_o |  | -0.018** | -0.003** |  | -0.018** | -0.003** |
| L_gdp_d |  | -0.019 | -0.004 |  | -0.02 | -0.004 |
| WTO_o |  | -0.1 | -0.019 |  | -0.1 | -0.019 |
| RTA_type |  | -0.001 | -0.0002 |  | -0.001 | -0.0003 |
| TRA_coverage |  | -0.007 | -0.001 |  | -0.007 | -0.001 |
| Intercept |  | 0.651 |  |  | 0.474 |  |

**Note**. ^1^ M.E indicates marginal effect and -- means do not apply.

*** is 99% significant; ** is 95% significant; * is 90% significant

**Table A. 3** Estimation results of fruits and vegetables products

|  |  | **Coefficients** | **M.E^1^** |  | **Coefficients** | **M.E^1^** |
| --- | --- | --- | --- | --- | --- | --- |
| China_EPU |  | 0.0001 | 0.00002 |  | -- | -- |
| Global_EPU |  | -- | -- |  | 0.001*** | 0.0003*** |
| L_tradevalue |  | -0.026*** | -0.006*** |  | -0.027*** | -0.006*** |
| Length |  | -0.119*** | -0.025*** |  | -0.119*** | -0.025*** |
| China_CPI |  | 0.0002 | 0.00003 |  | -0.002 | -0.0004 |
| Foodcapita |  | 0.027*** | 0.006*** |  | 0.037*** | 0.008*** |
| Urbanization |  | -0.03* | -0.006* |  | -0.043*** | -0.009*** |
| L_distance |  | 0.025 | 0.005 |  | 0.024 | 0.005 |
| Contiguity |  | -0.04 | -0.008 |  | -0.039 | -0.008 |
| L_gdp_o |  | -0.021*** | -0.004*** |  | -0.021*** | -0.004*** |
| L_gdp_d |  | 0.032 | 0.007 |  | 0.032 | 0.007 |
| WTO_o |  | 0.01 | 0.002 |  | 0.011 | 0.002 |
| RTA_type |  | -0.035 | -0.007 |  | -0.034 | -0.007 |
| TRA_coverage |  | 0.04* | 0.008* |  | 0.04* | 0.008* |
| Intercept |  | -0.893 |  |  | -1.055 |  |

**Note**. ^1^ M.E indicates marginal effect and -- means do not apply.

*** is 99% significant; ** is 95% significant; * is 90% significant

**Table A. 4** Estimation results of cereal products

|  |  | **Coefficients** | **M.E^1^** |  | **Coefficients** | **M.E^1^** |
| --- | --- | --- | --- | --- | --- | --- |
| China_EPU |  | 0.001* | 0.0001* |  | -- | -- |
| Global_EPU |  | -- | -- |  | 0.001 | 0.0001 |
| L_tradevalue |  | -0.018** | -0.003** |  | -0.018** | -0.003** |
| Length |  | -0.121*** | -0.023*** |  | -0.121*** | -0.023*** |
| China_CPI |  | 0.005 | 0.001 |  | 0.003 | 0.001 |
| Foodcapita |  | 0.027* | 0.005* |  | 0.02 | 0.004 |
| Urbanization |  | -0.06** | -0.011** |  | -0.04** | -0.008** |
| L_distance |  | -0.003 | -0.001 |  | -0.002 | -0.0004 |
| Contiguity |  | -0.266*** | 0.05*** |  | -0.265*** | -0.051*** |
| L_gdp_o |  | 0.001 | 0.0002 |  | 0.001 | 0.0002 |
| L_gdp_d |  | -0.011 | -0.002 |  | -0.011 | -0.002 |
| WTO_o |  | -0.115 | -0.022 |  | -0.112 | -0.022 |
| RTA_type |  | -0.063 | 0.012 |  | -0.064 | -0.012 |
| TRA_coverage |  | 0.071** | 0.014** |  | 0.071** | 0.014** |
| Intercept |  | 1.002 |  |  | 0.835 |  |

**Note**. ^1^ M.E indicates marginal effect and -- means do not apply.

*** is 99% significant; ** is 95% significant; * is 90% significant
